# The Non-Core Regions of Human Lysozyme Amyloid Fibrils Influence Cytotoxicity

**DOI:** 10.1016/j.jmb.2010.07.005

**Published:** 2010-10-08

**Authors:** Maria F. Mossuto, Anne Dhulesia, Glyn Devlin, Erica Frare, Janet R. Kumita, Patrizia Polverino de Laureto, Mireille Dumoulin, Angelo Fontana, Christopher M. Dobson, Xavier Salvatella

**Affiliations:** 1 Institute for Research in Biomedicine, Barcelona, Spain; 2 Department of Chemistry, University of Cambridge, Cambridge, UK; 3 CRIBI Biotechnology Centre, University of Padua, Padua, Italy; 4 Centre for Protein Engineering, University of Liege, Liege, Belgium; 5 ICREA, Barcelona, Spain

**Keywords:** ANS, 1-anilino-naphthalene-8-sulfonic acid, ATR-FTIR, attenuated total reflectance Fourier transform infrared, DMSO, dimethyl sulfoxide, GdnHCl, guanidine hydrochloride, GdnSCN, guanidine thiocyanate, DTNB, 5,5′-dithiobis-(2-nitrobenzoic acid), IR, infrared, MTT, 3-(4,5-dimethylthiazol-2-yl)-2,5-diphenyltetrazolium bromide, TCEP, tris(carboxyethyl)phosphine, TEM, transmission electron microscopy, ThT, thioflavin T, human lysozyme, amyloid fibrils, polymorphism, stability, toxicity

## Abstract

Identifying the cause of the cytotoxicity of species populated during amyloid formation is crucial to understand the molecular basis of protein deposition diseases. We have examined different types of aggregates formed by lysozyme, a protein found as fibrillar deposits in patients with familial systemic amyloidosis, by infrared spectroscopy, transmission electron microscopy, and depolymerization experiments, and analyzed how they affect cell viability. We have characterized two types of human lysozyme amyloid structures formed *in vitro* that differ in morphology, molecular structure, stability, and size of the cross-β core. Of particular interest is that the fibrils with a smaller core generate a significant cytotoxic effect. These findings indicate that protein aggregation can give rise to species with different degree of cytotoxicity due to intrinsic differences in their physicochemical properties.

## Introduction

Amyloid fibrils are non-covalent assemblies of proteins that form in the tissues of patients suffering from protein deposition diseases that include sporadic and transmissible neurodegenerative disorders^1,2^ as well as various non-neuropathic amyloidosis.^3,4^ It has been shown that non-fibrillar species transiently populated in the early phases of *in vitro* aggregation of many different proteins, including those not involved in disease, are more cytotoxic than the corresponding mature amyloid fibrils.^5–7^ This finding has led to the suggestion that the toxicity of these species is due to generic structural properties^1,8–10^ and adds to a significant body of evidence linking the onset of Alzheimer's and Parkinson's diseases with the formation of similar species in the brain of patients. Although non-fibrillar oligomers are the main focus of attention, recent studies have reported that, in some protein systems, amyloid fibrils can also produce a cytotoxic effect.^11–13^ In addition, it has been shown that prion diseases are caused by the propagation of infectious particles that carry all the information required to exhibit distinct phenotypic traits in identical hosts^14^ and are clearly fibrillar.^15^

Such observations raise the possibility that the cytotoxicity of protein aggregates in the biological milieu is not necessarily directly related to their oligomeric nature but, rather, to structural properties common to non-fibrillar and certain fibrillar aggregates. In contrast to highly evolved native structures, the structures of protein aggregates can be strongly influenced by pH, buffer components, protein concentration, and temperature;^16–22^ these findings have therefore led to intense research efforts aimed at establishing structure–activity relationships for protein aggregates. A particularly useful system to investigate such relationships is lysozyme, a protein with four disulfide bonds that is well characterized^23,24^ and forms amyloid deposits in patients suffering from familial lysozyme systemic amyloidosis,^25^ a disease that occurs when amyloidogenic mutations in the protein lead to the formation of partially unfolded amyloidogenic intermediates.^23,26,27^ By incubating lysozyme under various destabilizing conditions, we have produced fibrils differing in morphology, molecular structure, stability, and cytotoxicity. Our results illustrate that the energy landscape of aggregation is significantly more rugged than the folding landscape and, importantly for understanding the molecular basis of protein deposition disorders, that the pathogenic properties of the aggregates formed by lysozyme appear to be related to the fraction of sequence that is not included in the cross-β core of the fibrils.

## Results

### Amyloid fibrils formed under different conditions possess distinct cytotoxicities

**Fig. 1:**
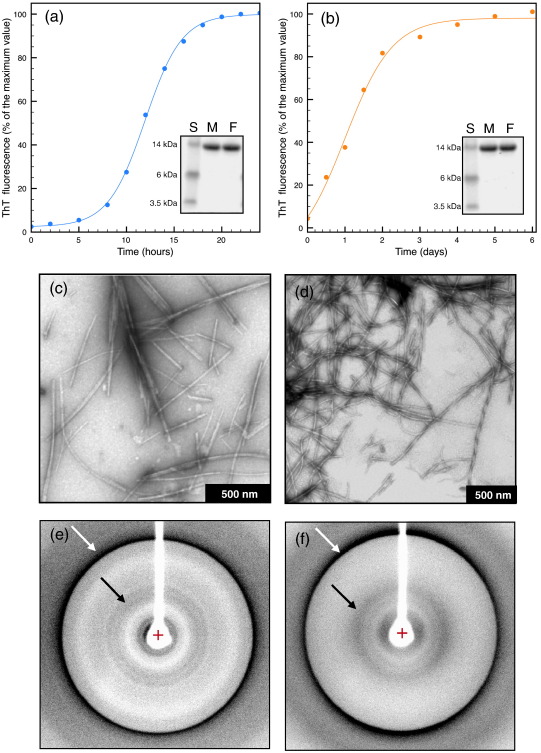
Amyloid morphology of lysozyme aggregates. The formation of amyloid fibrils from lysozyme at pH 7.5 (F^Phys^) (a) and at pH 2.0 (F^Acid^) (b) was monitored by the ThT binding assay. SDS-PAGE analysis (a and b, insets) of the formed fibrils, isolated by ultracentrifugation, confirmed that lysozyme remained intact in the fibrils. Standard molecular mass markers are shown in lanes S, aliquots of monomeric lysozyme are shown in lanes M, and aliquots of fibrillar lysozyme, previously dissolved in DMSO, are shown in lanes F. The TEM images of samples of F^Phys^ (c) and F^Acid^ (d) fibrils display, in both cases, a fibrillar and unbranched morphology. The X-ray fiber diffraction patterns of F^Phys^ (e) and F^Acid^ (f) fibrils correspond to the typical cross-β structure with meridional (white arrows) and equatorial reflections (black arrows).

Amyloid formation was performed under strongly destabilizing conditions, at pH 2.0, and under milder conditions, at pH 7.5. Since the formation of amyloid fibrils by human lysozyme is associated with the formation of partially folded species at the midpoint of thermal denaturation,^26^ aggregation at pH 2.0 was performed at 50 °C and that at pH 7.5 was performed at 60 °C, the lowest temperatures where intermediates could be detected.^26^ Aggregation of human lysozyme at pH 7.5 (Fig. 1a) was carried out at 60 °C, rather than at higher temperatures, to limit degradative reactions over the timescale of the experiment; at pH 2.0 (Fig. 1b), it was carried out at 50 °C, using seeding to eliminate the lag phase as the reduced time required for incubation under these conditions can prevent acid hydrolysis. The formation of amyloid fibrils, monitored by the thioflavin T (ThT) binding assay, took place at pH 7.5 (Fig. 1a) and at pH 2.0 (Fig. 1b) with the kinetics expected for non-seeded and seeded aggregation processes, respectively.

The transmission electron microscopy (TEM) images of the material isolated by ultracentrifugation (Fig. 1c and d) showed in both cases the presence of linear, unbranched fibrils with all the features expected in amyloid species but with differences in morphology. When prepared at pH 7.5 (Fig. 1c), the fibrils presented an average diameter of 10.7 ± 2.8 nm and tended to associate laterally (F^Phys^ fibrils), whereas the fibrils formed at pH 2.0 (Fig. 1d) presented a diameter of 6.5 ± 0.7 nm and were twisted (F^Acid^ fibrils). In neither case did the TEM images suggest the presence of non-fibrillar aggregates, and an analysis of the length distribution of the fibrillar material formed under acid and physiological conditions afforded no significant differences (Fig. S1). An analysis of the aggregates formed by human lysozyme using X-ray fiber diffraction yielded, despite some difficulties encountered in aligning the F^Phys^ fibrils, the two reflections characteristic of the cross-β structure (Fig. 1e and f): a meridional reflection at 4.8 Å and an equatorial reflection in the vicinity of 10 Å. The former, common to both morphologies, reports on the distance between the β-strands in each of the sheets of the amyloid protofilament and is a fundamental property, observed for all fibrils characterized to date.^4,28^ The latter, which reports on the spacing between each of the sheets,^28,29^ differed in the two types of fibrils: in F^Acid^ fibrils, the reflection was at 10.4 Å (Fig. 1f), whereas F^Phys^ fibrils showed a broad reflection centered at 10.3 Å (Fig. 1e).

**Fig. 2:**
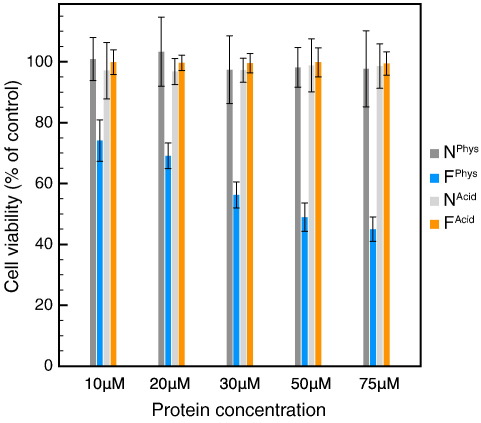
Effect of addition of lysozyme fibrils on the survival of SH-SY5Y neuroblastoma cells. SH-SY5Y cell viability in the presence of increasing concentrations (from 10 to 75 μM) of native human lysozyme at pH 2.0 (N^Acid^), F^Acid^ fibrils, native lysozyme at pH 7.5 (N^Phys^), and F^Phys^ fibrils.

The cytotoxicity of both types of fibrils was measured by studying their effect, at total protein concentrations ranging from 10 to 75 μM, on the viability of SH-SY5Y neuroblastoma cells using the 3-(4,5-dimethylthiazol-2-yl)-2,5-diphenyltetrazolium bromide (MTT) assay.^30^ In viable cells, MTT undergoes reduction by mitochondrial dehydrogenases (the succinate-tetrazolium reductase system) to yield insoluble formazan, which reports on the fraction of metabolically active cells. Figure 2 shows that SH-SY5Y cells were insensitive to native lysozyme and to F^Acid^ fibrils. By contrast, addition of the same quantities of the F^Phys^ fibrils reduced cell survival in a concentration-dependent manner. Moreover, the A11 generic anti-oligomer antibody developed by Kayed *et al.*^10^ did not react with the samples in the dot blot assay (data not shown), indicating that the cytotoxic effect described in Fig. 2 was not due to the presence of significant quantities of the non-fibrillar oligomeric species that are recognized by this antibody.^31^

### The cytotoxic F^Phys^ fibrils contain less cross-β structure than the inert F^Acid^ fibrils

**Fig. 3:**
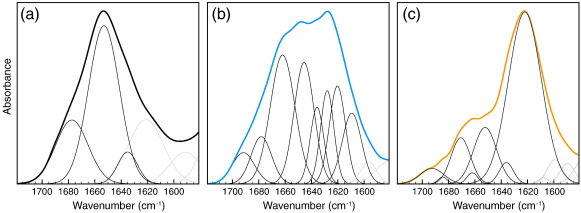
Structural characterization of native human lysozyme (N) (a) and of the F^Phys^ (b) and F^Acid^ (c) amyloid fibrils. The ATR-FTIR spectra are reported as thick lines, whereas thin lines correspond to the individual components obtained by curve fitting. Among the latter, black lines are assigned to signals corresponding to the main chain and gray lines are assigned to signals corresponding to side chains.

To explain the structural origin of such differences, we analyzed the amide I region (1580–1720 cm^− 1 ^) of the infrared (IR) spectrum measured using attenuated total reflectance Fourier transform infrared (ATR-FTIR) spectroscopy (Fig. 3). From a comparison of the spectra, it was evident that the secondary structure of fibrillar lysozyme was very different from that of the native protein and, most importantly, that the secondary structure of the toxic F^Phys^ fibrils was different from that of the inert F^Acid^ fibrils (Fig. 3 and Table 1). The FTIR bands obtained by deconvolution and curve fitting of the spectra were grouped into classes corresponding to secondary-structure elements: bands centered between 1620 and 1640 cm^− 1^ and between 1680 and 1700 cm^− 1 ^ were assigned to β-sheet, bands between 1640 and 1660 cm^− 1 ^ were assigned to random/α-helix, and bands between 1660 and 1680 cm^− 1^ were assigned to turns/loops. The results highlight that lysozyme undergoes profound changes in conformation during fibril formation; from its largely α/β native structure (25% β-sheet, 53% α-helix, and 22% loops and turns), lysozyme acquired 45% of β-sheet structure in the F^Phys^ fibrils and 75% in the F^Acid^ fibrils (Fig. 3 and Table 1). F^Phys^ fibrils contained 45% of β-sheet, 21% of random/α-helix, and 34% of turns/loops, whereas F^Acid^ fibrils contained 75% of β-sheet, 15% of random/α-helix, and 10% of turns/loops. These results clearly show that the observed polymorphism is associated with fundamentally different molecular conformations and, more importantly, that the cytotoxic effect of F^Phys^ fibrils was related to the amount of β structure.

**Fig. 4:**
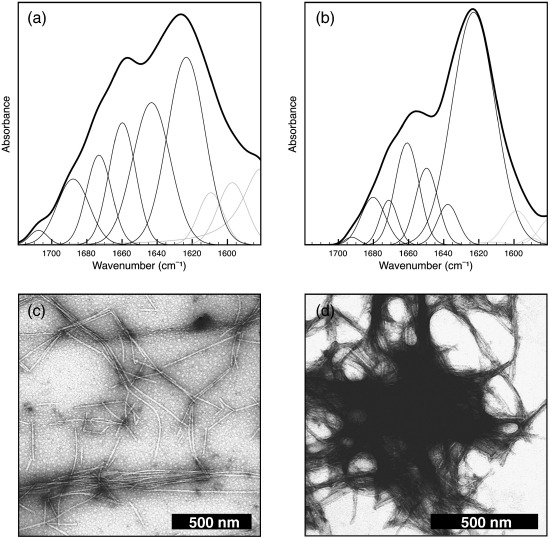
Properties of lysozyme fibrils after pH change. The pH of the solutions was changed by addition of minimal volumes of concentrated acidic or basic solutions. After 24 h at the altered pH, F^Phys^ (a and c) and F^Acid^ (b and d) fibrils were isolated by ultracentrifugation and their structure and morphology were characterized by FTIR (a and b) and TEM (c and d), respectively.

Since the cytotoxicity assay was performed, for the inert F^Acid^ fibrils, under conditions different from those of amyloid formation, it became important to determine whether their morphology had been affected by the pH change and, in general, whether the distinct morphologies interconverted upon changing conditions. The results showed that, after exposure to the altered pH by minimal additions of acid or base, the morphological and structural properties of the fibrils such as their diameter and secondary structure, verified respectively by TEM and FTIR, did not change significantly (Fig. 4 and Table 2). When F^Phys^ fibrils were incubated at pH 2.0, the β-sheet content remained constant at ca 45% (Fig. 4a and Table 2), the diameter of the fibrils did not change, and an increase in the number of individual fibrils—relative to that of the clusters seen at physiological pH (Figs. 1c and 4c)—was observed. F^Acid^ fibrils exposed to physiological pH showed instead a slightly decreased β-sheet content (71% instead of 75%) (Fig. 4b and Table 2), did not show detectable changes in diameter, and had a greater tendency to associate laterally to form bundles than at pH 2.0 (Figs. 1d and 4d). These changes observed in the extent of fibril association are likely to be due to electrostatic repulsions between individual protofilaments and fibrils due to the fact that the charge of lysozyme at acidic and physiological pH is + 21 and + 7, respectively.

Taken together, these results indicated that the pH at which the fibrils were grown affected their nature and morphology by modifying the conformation that the protein adopted within the fibrils, but that the kinetic barriers to reorganization of the fibrils once formed were sufficiently high to limit the effects of subsequent changes of solution conditions.

### The toxic F^Phys^ fibrils are less stable to depolymerization than the F^Acid^ fibrils

**Fig. 5:**
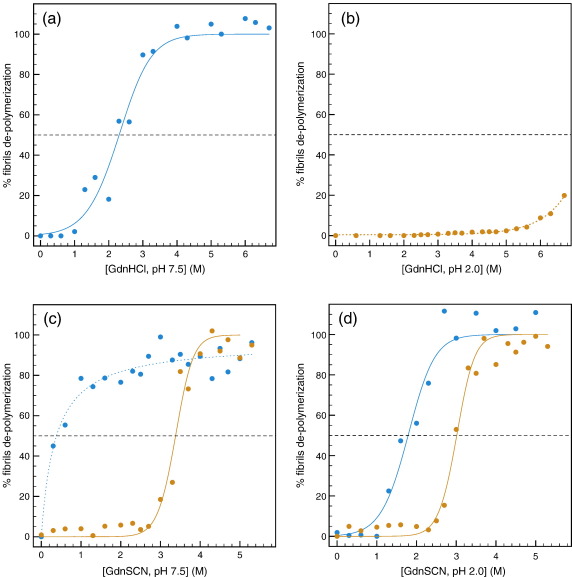
Conformational stability of F^Phys^ (blue) and F^Acid^ (orange) fibrils. The stability of the fibrils was measured by depolymerization experiments performed using GdnHCl at pH 7.5 (a) and at pH 2.0 (b) and using GdnSCN at pH 7.5 (c) and pH 2.0 (d). Continuous lines represent the best fits to a sigmoidal function.

In order to determine whether the existence of different morphologies with distinct degrees of toxicity had a kinetic or a thermodynamic origin, we assessed the stability of the fibrils by measuring their resistance to depolymerization. The experiments were carried out by incubating aliquots of fibrils in solutions containing increasing concentrations of chaotrope and then measuring the equilibrium concentration of soluble monomeric protein present in the supernatant. Plots of the fraction of soluble protein released from the amyloid fibrils at different concentrations of chaotrope are presented in Fig. 5. Initial studies carried out using guanidine hydrochloride (GdnHCl) provided evidence that the F^Phys^ fibrils were remarkably less stable than the F^Acid^ fibrils; the former show a midpoint of depolymerization at ca 2.5 M GdnHCl (Fig. 5a), whereas the depolymerization of the latter was incomplete even in a saturated solution of GdnHCl (Fig. 5b).

To obtain comparable midpoints of depolymerization, we repeated the procedure using a significantly stronger chaotrope, guanidine thiocyanate (GdnSCN), and the results of these experiments yielded 0.5  and 3 M as the midpoints of depolymerization of the F^Phys^ and F^Acid^ fibrils, respectively (Fig. 5c and d). Since the stability of fibrils has been attributed primarily to the large number of hydrogen-bonding interactions that are established between individual protein molecules in the cross-β structure,^32,33^ these results are in agreement with the structural characterization carried out by IR spectroscopy, which showed that the inert F^Acid^ fibrils were richer in β-sheet structure (75% β-sheet for F^Acid^ *versus* 45% for F^Phys^) and were therefore likely to contain significantly more inter-strand hydrogen bonds than the toxic F^Phys^ fibrils.

To compare the stabilities of the two forms under the same conditions, and taking advantage of the fact that the fibrils did not interconvert at a measurable rate (Fig. 4), we repeated the depolymerization experiments for the F^Phys^ fibrils at pH 2.0 and for the F^Acid^ fibrils at pH 7.5. The results showed that F^Acid^ fibrils exposed to pH 7.5 were still more stable (midpoint of depolymerization at ca 3.4 M GdnSCN) than F^Phys^ fibrils exposed to pH 2.0 (midpoint of depolymerization at ca 1.8 M GdnSCN). The results (Fig. 5) confirmed that the F^Phys^ fibrils did not represent the most stable configuration of the lysozyme aggregates under a given set of conditions but were instead trapped in a kinetically stable configuration that contained a lower number of stabilizing hydrogen-bonding interactions than the F^Acid^ fibrils.

### Nature of the non-core regions of lysozyme fibrils

**Fig. 6:**
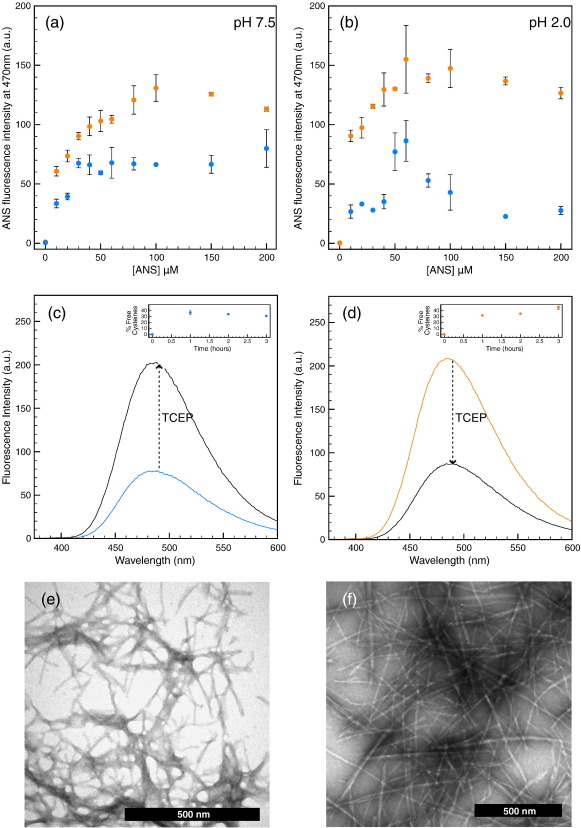
ANS binding on F^Phys^ (blue) and F^Acid^ (orange) fibrils measured at pH 7.5 (a) and pH 2.0 (b). The ANS fluorescence intensity measured at 470 nm is plotted as a function of the ANS concentration. Protein concentration was 5 μM in all cases. (c and d) ANS binding on F^Phys^ (blue) and F^Acid^ (orange) fibrils isolated after 1 h reduction of the disulfide bonds; the concentration of ANS in these experiments was 100 μM. (c and d, insets) Time dependence of the percentage of free cysteines in the fibrillar samples after reduction with TCEP. (e and f) TEM images of the F^Phys^ and F^Acid^ fibrils, respectively, after 1 h of reduction with TCEP.

To gain further insight into the nature of the non-core regions of both types of fibrils, we measured their ability to bind 1-anilino-naphthalene-8-sulfonic acid (ANS), a dye that displays an increase in fluorescence emission intensity and a blue shift of its wavelength of maximum emission upon binding clusters of hydrophobic side chains in non-native proteins such as molten globules.^7,34–36^ The results of these experiments, presented in Fig. 6, revealed that, under both pH conditions studied in this work (pH 7.5 and pH 2.0), the fluorescence intensity of ANS in the presence of the inert F^Acid^ fibrils was higher than that in the presence of the cytotoxic F^Phys^ fibrils. Given that the more stable F^Acid^ fibrils have a larger cross-β core than the F^Phys^ fibrils (75% of β structure for F^Acid^ *versus* 45% for F^Phys^), this result indicated that the hydrophobic side chains of the non-core regions of the latter were, in spite of their larger number, less accessible than those of the non-core regions of the former, presumably due to the formation of local structure or to differences in the packing of the protofilaments.

To investigate the origin of this lack of accessibility, and by taking advantage of the presence of eight Cys residues involved in the formation of four disulfide bonds, we exposed preparations of both types of fibrils to the reducing agent tris(carboxyethyl)phosphine (TCEP). Using Ellman's assay, we found that, after 1 h of reaction time, ca 30–40% of the Cys residues of both types of fibrils were reduced (Fig. 6c and d, insets), that the fraction of reduced Cys residues did not change significantly with time, and, using TEM, that the morphology of the fibrils was unaffected by the reaction (Fig. 6e and f). That both types of fibrils show the same reactivity against TCEP suggested that the differences in ANS binding were due to the presence of local structure rather than to an inability of this dye to access the non-core regions of F^Phys^ fibrils. This was confirmed by an analysis of the ANS binding ability of the fibrils after 1 h in the presence of TCEP, which yielded, in contrast with the results obtained prior to reduction, that the fluorescence intensity of ANS was higher in the presence of F^Phys^ fibrils than in the presence of F^Acid^ fibrils (Fig. 6c and d). As expected from the relative size of the non-core regions of these two types of fibrils, these results indicated that the partially reduced F^Phys^ amyloid fibrils display a larger number of clustered hydrophobic side chains than the partially reduced F^Acid^ fibrils and suggested the presence of local structure, stabilized by the native disulfide bonds of lysozyme, in the non-core regions of the fibrils formed under physiological pH. These results are in agreement with the recent observation that the cytotoxicity of non-fibrillar oligomers formed by the protein HypF-N correlates with ANS binding.^7^

## Discussion

In contrast to globular proteins, which have evolved to fold under physiological conditions into a single well-defined structure, each protein sequence can form *in vitro* a wide range of distinct aggregates depending on conditions of incubation.^16^ Such polymorphism affects the biological properties of protein aggregates; in fact, small metastable oligomers, rather than mature fibrils, are thought to be the toxic species in neurodegenerative diseases^1,5^ and amyloid polymorphs are at the basis of species barriers and strains in prion diseases.^37,38^ Since the fate of the aggregation process can vary depending on the tissue in which it occurs, on the cellular and subcellular localization of the protein, and on environmental conditions,^18,39,40^ it is important to increase our understanding of how these species acquire the characteristics that lead to disease.

We have found that the existence of different morphologies in the fibrillar material formed by human lysozyme *in vitro* is due to fundamental differences in the conformation of the protein within the amyloid structure. Toxic fibrils formed at physiological pH (F^Phys^) have a lower β-sheet content (Fig. 3) and are less stable to depolymerization (Fig. 5) than those formed at acidic pH (F^Acid^), which are not detactably toxic in our essay. Such polymorphism is different from that of fibrils formed from polypeptides devoid of cooperatively folded structure such as the GNNQQNY and NNQQ peptides from the yeast prion protein Sup35,^41^ α-synuclein,^22^ and Aβ,^19^ where it is instead probably due to differences in the orientation of the protofilaments and the inter-digitation of side chains, which lead to fibrils that, contrary to what we have observed, have very similar stabilities.^19^ In the case of human lysozyme, it is instead reminiscent of that displayed by the yeast prion protein Sup35, where fibrils formed at 4  and 37 °C presented distinct morphologies and biophysical properties,^37^ or by the fungal prion HET-s, where fibrils formed under pH 3.0 and pH 7.0 yielded very different NMR spectra and infectivity.^42^ The polymorphism that we observed and characterized in this work occurs at the molecular level, that is, in the way lysozyme molecules fold in the different amyloid states. These fundamental differences in the properties of the protein embedded in the fibrils can, however, have consequences with regard to the morphology, shape and diameter, of the two types of fibrils (Fig. 1). Variations in the number, arrangement, and diameter of protofilaments could in fact arise from the way the lysozyme molecules in the fibrillar context guide the hierarchical supra-molecular assembly of the fibrils.

Our results provide new insights into polymorphism by experimentally illustrating that the energy landscape of aggregation is more rugged than the evolved folding landscape of globular proteins. Specifically, we have found that the F^Phys^ fibrils formed by lysozyme, which are intrinsically less stable than the F^Acid^ fibrils, remain in essentially the same conformational state when the pH is changed to that in which the F^Acid^ fibrils form (Figs. 4 and 5). This finding provides clear evidence for the presence of at least two local minima on the energy landscape separated by a kinetic barrier sufficiently high to prevent the interconversion of these two species *in vitro*. This lack of ability of the aggregation process, under physiological pH, to achieve the most stable form of amyloid fibrils is similar to that observed in the yeast prion protein Sup35^37^ and in the polyQ protein huntingtin, where aggregation reactions carried out at 37 °C led to amyloid fibrils that had a higher thermal stability than those formed at 4 °C, presumably because the kinetic barrier that led to the most stable fibrillar morphology was too high to be overcome at the lower temperature.^43^ While amyloid polymorphism in the aggregation of intrinsically disordered proteins and peptides^19,41^ is often found to have a thermodynamic origin, our results with lysozyme show that it can also have a kinetic origin and be a direct consequence of the ruggedness of the energy landscape of the aggregating protein caused, at least in part, by the presence of four disulfide bridges that remain formed in the amyloid fibrils.

The study of polymorphism in protein aggregates is evidently important for our understanding of the physical principles that govern fibril formation but is also very timely due to recent suggestions that the degree of cross-β structure present in non-fibrillar oligomers is related to their cytotoxicity and, potentially, to their ability to trigger neurodegenerative disorders.^44^ This structure–activity relationship^5,45^ is, however, very difficult to verify at equilibrium due to the transient nature of the metastable species populated during fibril formation.^7,46,47^ Establishing structure–activity relationships in amyloid structures is also important because the phenotypic traits associated with prion diseases are due to the existence of different amyloid morphologies, formed by the same sequence and presenting different physical and structural properties, the so-called prion strains.^14^ The availability of kinetically stable amyloid morphologies of lysozyme with different degrees of cross-β structure, which can be formed under controlled and reproducible conditions, has given us a unique opportunity to determine whether the presence of large non-core regions in the fibrillar material is related to cytotoxicity. The results that we have obtained show that F^Phys^ fibrils, which are less ordered in the cross-β structure, generate a significant cytotoxic effect while the highly organized F^Acid^ fibrils have a small or negligible effect on cell viability. These findings suggest that even aggregates with all the hallmarks of amyloid fibrils, but provided with extensive non-core regions, can give rise to cytotoxicity; they, therefore, support the hypothesis that the cytotoxicity of certain protein aggregates is related to their structural and dynamic properties and, in particular, to the presence of substantial non-core regions.

It is possible to invoke a number of mechanisms by which the presence of non-core regions in amyloid fibrils could give rise to cytotoxicity. The most straightforward of them would involve interactions of the non-core regions of the fibrils with the cell membrane, in the extracellular environment or, inside the cell, with components of the cellular machinery.^7,9,10^ It is however possible that other phenomena play a role in triggering cytotoxicity because the size of the cross-β core defines important mechanical properties of amyloid fibrils such as their propensity to fragment.^6,48–51^ Our observation that the size distributions of both fibrillar morphologies are similar suggests that interactions between fibrils and cells could underlie cytotoxicity, but further experiments will be required to determine the mechanism by which F^Phys^ fibrils decrease cell viability.

## Conclusion

Understanding why protein deposition leads to disease will be crucial in developing therapeutic approaches aimed at preventing or curing increasingly prevalent disorders such as Alzheimer's and Parkinson's diseases. The results that we obtained with human lysozyme clearly indicate that the presence of kinetic traps in the energy landscape of the aggregated protein can lead to the formation of species that are only partially structured in the cross-β conformation. That these fibrillar species lead to cytotoxicity strongly suggests that this phenomenon is associated with the presence of regions of the polypeptide chain that are not structured in the cross-β conformation either directly or as a result of their effect on fibril stability.

## Materials and Methods

### Materials

Human lysozyme was expressed and purified as previously described.^52^ ANS and thioflavin-T (ThT) were purchased from Sigma-Aldrich (St. Louis, MO). All other chemicals were of analytical reagent grade and were obtained from Sigma-Aldrich.

### Methods

#### Formation of human lysozyme amyloid fibrils at pH 2.0 and pH 7.5

Fibrils were prepared at pH 7.5 by dissolving lysozyme in 50 mM Na_2_HPO_4_ at a concentration of 0.7 mM and stirring the solution at 60 °C for 1 day. Formation of fibrils at pH 2.0 was induced by seeding a 1 mM lysozyme solution in 10 mM HCl, pH 2.0, at a ratio of 2% (w/w) with aliquots of fibrils preformed at pH 2.0, 50 °C, in the absence of seeding. The suspension was then left at 50 °C and stirred for up to 6 days. Samples of fibrils, isolated by ultracentrifugation (90,000 rpm, 4 °C, 1.5 h), were characterized by ThT binding and TEM. The insoluble material was dissolved in 95% dimethyl sulfoxide (DMSO) and analyzed by SDS-PAGE, reverse-phase high-performance liquid chromatography, and mass spectrometry.

#### Optical spectroscopy

Protein concentrations were evaluated from absorption measurements at 280 nm on a single-beam Cary 400 Scan spectrophotometer (Varian, Palo Alto, CA, USA). The extinction coefficient of full-length human lysozyme^53^ at 280 nm was 36,940 cm^− 1 ^ M^− 1 ^.

Fluorescence measurements were carried out on a Varian model Cary Eclipse spectrofluorimeter in a temperature-controlled cell holder, utilizing a 2 mm × 10 mm path-length cuvette. For each measurement, a protein concentration of 2.4 μM was used. ThT binding was monitored by exciting the sample at 440 nm and recording the emission fluorescence spectrum from 450 to 600 nm. For each measurement, 25 μl of a 2.5 mM ThT stock solution prepared in 10 mM phosphate buffer (pH 7.0) containing 150 mM NaCl was added to a volume of fibrils corresponding to 60 μg and a volume of 1.5 ml was reached with the phosphate buffer. The fluorescence emission of ThT at 485 nm was fitted to a four-parameter sigmoidal curve using Sigma Plot (Systat Software Inc., California, USA) for each aggregation reaction. For ANS titration, aliquots of ANS from a stock solution in water were added to the isolated fibrils, to a final ANS concentration ranging from 0 to 200 μM. The final protein concentration was 5 μM in all cases. The spectra were immediately acquired at 20 °C, using an excitation wavelength of 350 nm and an emission range from 380 to 700 nm. The difference between the resulting fluorescence intensity at 470 nm and that measured with only ANS in the absence of protein was used as the effective bound ANS fluorescence. The extinction coefficient at 350 nm of ANS was 4950 cm^− 1^  M^− 1^.^35^ Each assay was repeated three times.

#### Reduction of fibrillar disulfide bonds

To follow the reaction of lysozyme fibrils with the reducing agent TCEP, we resuspended amyloid fibrils isolated by ultracentrifugation in 10 mM HCl, pH 2.0, containing 10 molar excess of TCEP over the number of cysteines in the sample at 25 °C. From the reaction tube, 100 μl was taken at given time points (1–3 h) and ultracentrifuged (90,000 rpm, 4 °C, 45 min), and the resulting pellet was washed and resuspended in 10 mM HCl, pH 2.0. Aliquots of these samples were taken for the 5,5′-dithiobis-(2-nitrobenzoic acid) (DTNB) assay, ANS titration, and TEM. Working at acidic pH allowed the disulfide bonds to be reduced with TCEP^54^ while maintaining the fibrillar morphologies as well as the oxidation state of the cysteine side chains. Free thiol groups in fibrillar samples were determined spectrophotometrically at 412 nm using the DTNB assay.^55^ Fibrillar samples (7 μM) were incubated for 15 min at 25 °C with 0.17 mM DTNB in 0.1 M potassium phosphate buffer and 1 mM ethylenediaminetetraacetic acid (EDTA), pH 8.0. The values of absorbance at 412 nm were compared with a calibration curve obtained with known amounts of fully reduced lysozyme. Each assay was repeated three times.

#### Cellular viability monitored by the MTT assay

The cytotoxicity of fibrils was determined using the MTT assay. Neuroblastoma SH-SY5Y cells were cultured in Dulbecco's modified Eagle's medium supplemented with 10% (v/v) fetal bovine serum, 1% glutamine, and antibiotics in a 5% CO_2_ humidified atmosphere at 37 °C. They were plated (2000 cells/well) in a 96-well plate and incubated for 24 h. The incubation medium was then removed, and preparations of protein diluted in Dulbecco's modified Eagle's medium were added at concentrations between 10 and 75 μM and incubated for 48 h at 37 °C. After 46 h, MTT was added to a final concentration of 0.5 mg/ml and the cells were incubated for two additional hours. The medium was then removed, and 200 μl of 2-propanol was added in order to dissolve the purple formazan produced. The absorbance was then measured at 570 nm after 45 min and cell viability percentages were determined by dividing the absorbance value of cells treated with protein by that of untreated cells.

#### SDS-PAGE analysis

Fibrillar samples, previously dissolved in DMSO, were analyzed by SDS-PAGE using 4–12% 2-[bis(2-hydroxyethyl)amino]-2-(hydroxymethyl)propane-1,3-diol NuPAGE gels (Invitrogen UK) in 4-morpholineethanesulfonic acid buffer under reducing conditions. Gels were stained using Coomassie brilliant blue.

#### Bradford assay

Protein solutions (20 μl) were diluted 1:25 into the Bradford solution (Sigma-Aldrich) and left for 30 min at 25 °C, and the measured absorbance at 595 nm was compared with a calibration curve previously established using standard lysozyme solutions.

#### Transmission electron microscopy

Samples were applied to Formvar-coated nickel grids, stained with 2% (w/v) uranyl acetate solution, and viewed in a Phillips CEM100 transmission electron microscope operating at 80 kV. TEM images were analyzed using the ImageJ software.

#### X-ray fiber diffraction

Amyloid fibril samples were prepared for X-ray diffraction analysis using a modification of the stretch-frame method.^56^ In this modification, the distance between capillary tubes is not changed during the production of the semi-aligned, dry stalk. A  10-μl droplet of fibril suspension was placed between two horizontal wax-plugged glass capillary tubes of 1 mm diameter held approximately 2 mm apart. Upon drying of the droplet to approximately a quarter of its initial volume, a further 5 μl of fibril suspension was added and the process was repeated a further three times. Subsequently, the droplet was left to dry completely in air. All scattering patterns were obtained on a crystallography beamline at the Department of Biochemistry of the University of Cambridge. X-rays with a wavelength of 1.54 Å were produced by a rotating copper anode and collimated and focused by Osmic Max-flux optics, and images were acquired on a Marr image plate. The sample–detector distance was 300 mm, and data acquisition times were typically 15 min. Images were analyzed and radially integrated to generate 1D scattering patterns using Fit2D† to obtain accurate reflection positions.

#### pH change of fibrillar samples

The pH of fibril samples was adjusted to the desired value by adding minimal volumes (1–4% v/v) of concentrated HCl and NaOH solutions. The final pH value was measured with a pH meter. After 24 h at room temperature, the pH-swapped fibril samples were analyzed by using ultracentrifugation (90,000 rpm, 90 min, 4 °C), TEM, IR spectroscopy, and depolymerization experiments.

#### Attenuated total reflectance Fourier transform infrared spectroscopy

For secondary-structure analysis of fibrils, samples were analyzed in a Bruker BioATRCell II using a Bruker Equinox 55 FTIR spectroscopy spectrometer (Bruker Optics Limited, UK) equipped with a liquid-nitrogen-cooled mercury cadmium telluride detector and a silicon internal reflection element. For each spectrum, 256 interferograms were coadded at 2 cm^– 1^ resolution, and the buffer background was independently measured and subtracted from each protein spectrum, before curve fitting of the amide I region (1720–1580 cm^− 1 ^). Calculation of the second derivatives was used to identify peak maxima. Using this information, we then fitted the raw spectra to a series of Gaussian peaks with the identified absorbance maxima using an iterative curve-fitting procedure performed in Origin8 (OriginLab Corporation, Massachusetts, USA).

#### Measurement of conformational stability of fibrils

To measure the stability of fibrillar lysozyme, we diluted aliquots of fibrils into buffered solutions containing increasing concentrations of GdnHCl or GdnSCN, and after 72 h at 25 °C, the samples were ultracentrifuged (90,000 rpm, 20 °C, 45 min). The concentration of lysozyme in the supernatant was measured by recording the absorbance at 280 nm or by using the Bradford assay. The depolymerization curves were obtained by plotting the fraction of lysozyme released from the fibrils at various concentrations of denaturant. The time dependence of fibril depolymerization was studied by following the amount of lysozyme released by fibrils after dilution into a given concentration of chaotropic agent, in a time period from 15 min to 6 days. Samples of fibrils were diluted into buffered solution containing high concentration of GdnHCl or GdnSCN: at given time points, aliquots of these samples were ultracentrifuged and the concentration of protein in the supernatant was measured. The results showed that after a very fast initial release (2 h), the system reached equilibrium (Fig. S2).

## Figures and Tables

**Table 1:** Secondary-structure content of native and fibrillar human lysozyme as determined by curve fitting of the ATR-FTIR spectra shown in Fig. 3

Assignment	N		F^Phys^		F^Acid^	
	cm^− 1 ^a	%^b^	cm^− 1 ^a	%^b^	cm^− 1 ^a	%^b^
β-Sheet			1620/1628	25.3 ± 3.0	1622	67.5 ± 5.0
	1635	24.6 ± 3.0	1636	6.4 ± 2.0	1636	2.5 ± 1.5
Random/α-helix	1653	53.0 ± 2.0	1646	21.2 ± 3.0	1652	14.5 ± 0.5
Turns/loops	1677	22.4 ± 1.5	1662	33.4 ± 5.0	1662/1671	10.5 ± 0.4
β-Sheet			1680	3.4 ± 3.0	1684	0.5 ± 0.3
			1692	10.3 ± 3.0	1693	4.5 ± 0.5

a Peak position of the amide I band components, as deduced from the second-derivative spectra.

b Percentage area of the amide I band components, as obtained by integrating the area under each deconvoluted band. The error intervals were calculated from the percentages obtained in three separate experiments; the areas corresponding to side-chain contributions located at 1580–1610 cm^− 1 ^ were not considered.

**Table 2:** Secondary-structure content of lysozyme fibrils after pH change as determined by curve fitting of ATR-FTIR spectra shown in Fig. 4

Assignment	F^Phys^ at pH 2.0		F^Acid^ at pH 7.5	
	cm^− 1 ^a	%^b^	cm^− 1 ^a	%^b^
β-Sheet	1623	34.9 ± 5.0	1622	63.6 ± 5.0
Random/α-helix	1642	26.8 ± 4.0	1652	10.0 ± 2.0
Turns/loops	1662/1672	27.5 ± 3.0	1662/1672	18.7 ± 1.0
β-Sheet	1687	9.0 ± 3.0	1682	7.1 ± 2.0
	1702	1.8 ± 3.0	1693	0.6 ± 3.0

a Peak position of the amide I band components, as deduced by the second-derivative spectra.

b Percentage area of the amide I band components, as obtained by integrating the area under each deconvoluted band. The error intervals were calculated from the percentages obtained in three separate experiments; the areas corresponding to side-chain contributions located at 1580–1610 cm^− 1 ^ were not considered.

## Appendix A

Fig. S1 Average length and length distribution of the F^Phys^ and F^Acid^ fibrils as analyzed from the TEM micrographs; N_fibril_ refers to the number of fibrils considered. Fibril solutions were examined using TEM by negative staining. Diluted samples were deposited onto Formvar-coated copper grids (400 mesh) (Agar Scientific, Stansted, UK). Filter paper was used to remove the excess of sample after 45 s, before adding a droplet of staining solution [2% (w/v) uranyl acetate]. After another 45 s, the excess was removed and the sample was left to air-dry. The micrographs were taken using a Transmission Electron Microscope JEOL 1010 operating at 80 kV. Images were taken with a Megaview III camera and digitized with the software AnalySIS (Soft Imaging System). TEM images were analyzed using ImageJ. From the TEM pictures, fibrils were counted and their length was measured to produce the size distribution of the fibrils. To obtain reliable statistics, between 150 and 230 fibrils were analyzed for each sample.

Fig. S2 Depolymerization rate of F^Phys^ (blue) and F^Acid^ (orange) fibrils in the presence of guanidine salts. The reactions were initiated by diluting fibrillar samples into buffered solutions of the chaotropic agent. At each incubation time, the reaction mixture was analyzed by ultracentrifugation and the Bradford assay. Each point represents the average of three independent experiments.

## Footnotes

† http://www.esrf.fr/computing/scientific/FIT2D

## References

[1] Lansbury P., Lashuel H. (2006). A century-old debate on protein aggregation and neurodegeneration enters the clinic. Nature.

[2] Haass C., Selkoe D.J. (2007). Soluble protein oligomers in neurodegeneration: lessons from the Alzheimer's amyloid β-peptide. Nat. Rev. Mol. Cell Biol..

[3] Merlini G., Bellotti V. (2003). Molecular mechanisms of amyloidosis. N. Engl. J. Med..

[4] Chiti F., Dobson C.M. (2006). Protein misfolding, functional amyloid, and human disease. Annu. Rev. Biochem..

[5] Bucciantini M., Giannoni E., Chiti F., Baroni F., Formigli L., Zurdo J. (2002). Inherent toxicity of aggregates implies a common mechanism for protein misfolding diseases. Nature.

[6] Silveira J.R., Raymond G.J., Hughson A.G., Race R.E., Sim V.L., Hayes S.F., Caughey B. (2005). The most infectious prion protein particles. Nature.

[7] Campioni S., Mannini B., Zampagni M., Pensalfini A., Parrini C., Evangelisti E. (2010). A causative link between the structure of aberrant protein oligomers and their toxicity. Nat. Chem. Biol..

[8] Lashuel H.A., Hartley D., Petre B.M., Walz T., Lansbury P.T. (2002). Neurodegenerative disease: amyloid pores from pathogenic mutations. Nature.

[9] Stefani M., Dobson C.M. (2003). Protein aggregation and aggregate toxicity: new insights into protein folding, misfolding diseases and biological evolution. J. Mol. Med..

[10] Kayed R., Head E., Thompson J.L., McIntire T.M., Milton S.C., Cotman C.W., Glabe C.G. (2003). Common structure of soluble amyloid oligomers implies common mechanism of pathogenesis. Science.

[11] Meyer-Luehmann M., Spires-Jones T.L., Prada C., Garcia-Alloza M., de Calignon A., Rozkalne A. (2008). Rapid appearance and local toxicity of amyloid-β plaques in a mouse model of Alzheimer's disease. Nature.

[12] Engel M.F., Khemtémourian L., Kleijer C.C., Meeldijk H.J., Jacobs J., Verkleij A.J. (2008). Membrane damage by human islet amyloid polypeptide through fibril growth at the membrane. Proc. Natl Acad. Sci. USA.

[13] Xue W.F., Hellewell A.L., Gosal W.S., Homans S.W., Hewitt E.W., Radford S.E. (2009). Fibril fragmentation enhances amyloid cytotoxicity. J. Biol. Chem..

[14] Falsig J., Nilsson K.P., Knowles T.P., Aguzzi A. (2008). Chemical and biophysical insights into the propagation of prion strains. HFSP J..

[15] Aguzzi A., Heikenwalder M., Polymenidou M. (2007). Insights into prion strains and neurotoxicity. Nat. Rev., Mol. Cell Biol..

[16] Jiménez J., Nettleton E.J., Bouchard M., Robinson C.V., Dobson C.M., Saibil H. (2002). The protofilament structure of insulin amyloid fibrils. Proc. Natl Acad. Sci. USA.

[17] Zurdo J., Guijarro J.I., Jiménez J.L., Saibil H.R., Dobson C.M. (2001). Dependence on solution conditions of aggregation and amyloid formation by an SH3 domain. J. Mol. Biol..

[18] Petkova A.T., Leapman R.D., Guo Z., Yau W.M., Mattson M.P., Tycko R. (2005). Self-propagating, molecular-level polymorphism in Alzheimer's β-amyloid fibrils. Science.

[19] Meinhardt J., Sachse C., Hortschansky P., Grigorieff N., Fändrich M. (2009). Aβ(1–40) fibril polymorphism implies diverse interaction patterns in amyloid fibrils. J. Mol. Biol..

[20] Gosal W.S., Morten I.J., Hewitt E.W., Smith D.A., Thomson N.H., Radford S.E. (2005). Competing pathways determine fibril morphology in the self-assembly of β(2)-microglobulin into amyloid. J. Mol. Biol..

[21] Krishnan R., Lindquist S.L. (2005). Structural insights into a yeast prion illuminate nucleation and strain diversity. Nature.

[22] Heise H., Hoyer W., Becker S., Andronesi O.C., Riedel D., Baldus M. (2005). Molecular-level secondary structure, polymorphism, and dynamics of full-length α-synuclein fibrils studied by solid-state NMR. Proc. Natl Acad. Sci. USA.

[23] Dumoulin M., Kumita J.R., Dobson C.M. (2006). Normal and aberrant biological self-assembly: insights from studies of human lysozyme and its amyloidogenic variants. Acc. Chem. Res..

[24] Merlini G., Bellotti V. (2005). Lysozyme: a paradigmatic molecule for the investigation of protein structure, function and misfolding. Clin. Chim. Acta.

[25] Pepys M.B., Hawkins P.N., Booth D.R., Vigushin D.M., Tennent G.A., Soutar A.K. (1993). Human lysozyme gene mutations cause hereditary systemic amyloidosis. Nature.

[26] Booth D.R., Sunde M., Bellotti V., Robinson C.V., Hutchinson W.L., Fraser P.E. (1997). Instability, unfolding and aggregation of human lysozyme variants underlying amyloid fibrillogenesis. Nature.

[27] Dumoulin M., Last A.M., Desmyter A., Decanniere K., Canet D., Larsson G. (2003). A camelid antibody fragment inhibits the formation of amyloid fibrils by human lysozyme. Nature.

[28] Sunde M., Serpell L.C., Bartlam M., Fraser P.E., Pepys M.B., Blake C.C. (1997). Common core structure of amyloid fibrils by synchrotron X-ray diffraction. J. Mol. Biol..

[29] Fändrich M., Dobson C.M. (2002). The behaviour of polyamino acids reveals an inverse side chain effect in amyloid structure formation. EMBO J..

[30] Mosmann T. (1983). Rapid colorimetric assay for cellular growth and survival: application to proliferation and cytotoxicity assays. J. Immunol. Methods.

[31] Glabe C.G. (2008). Structural classification of toxic amyloid oligomers. J. Biol. Chem..

[32] Sunde M., Blake C. (1997). The structure of amyloid fibrils by electron microscopy and X-ray diffraction. Adv. Protein Chem..

[33] Knowles T.P., Fitzpatrick A.W., Meehan S., Mott H.R., Vendruscolo M., Dobson C.M., Welland M.E. (2007). Role of intermolecular forces in defining material properties of protein nanofibrils. Science.

[34] Semisotnov G.V., Rodionova N.A., Razgulyaev O.I., Uversky V.N., Gripas' A.F., Gilmanshin R.I. (1991). Study of the “molten globule” intermediate state in protein folding by a hydrophobic fluorescent probe. Biopolymers.

[35] Cardamone M., Puri N.K. (1992). Spectrofluorimetric assessment of the surface hydrophobicity of proteins. Biochem. J..

[36] Kremer I., Rietschel M., Dobrusin M., Mujaheed M., Murad I., Blanaru M. (2000). No association between the dopamine D3 receptor Bal I polymorphism and schizophrenia in a family-based study of a Palestinian Arab population. Am. J. Med. Genet..

[37] Tanaka M., Chien P., Naber N., Cooke R., Weissman J.S. (2004). Conformational variations in an infectious protein determine prion strain differences. Nature.

[38] Tanaka M., Collins S.R., Toyama B.H., Weissman J.S. (2006). The physical basis of how prion conformations determine strain phenotypes. Nature.

[39] Myers S.L., Jones S., Jahn T.R., Morten I.J., Tennent G.A., Hewitt E.W., Radford S.E. (2006). A systematic study of the effect of physiological factors on β2-microglobulin amyloid formation at neutral pH. Biochemistry.

[40] Bellotti V., Chiti F. (2008). Amyloidogenesis in its biological environment: challenging a fundamental issue in protein misfolding diseases. Curr. Opin. Struct. Biol..

[41] Sawaya M.R., Sambashivan S., Nelson R., Ivanova M.I., Sievers S.A., Apostol M.I. (2007). Atomic structures of amyloid cross-β spines reveal varied steric zippers. Nature.

[42] Wasmer C., Soragni A., Sabaté R., Lange A., Riek R., Meier B. (2008). Infectious and noninfectious amyloids of the HET-s(218–289) prion have different NMR spectra. Angew. Chem. Int. Ed..

[43] Nekooki-Machida Y., Kurosawa M., Nukina N., Ito K., Oda T., Tanaka M. (2009). Distinct conformations of in vitro and in vivo amyloids of huntingtin-exon1 show different cytotoxicity. Proc. Natl Acad. Sci. USA.

[44] Karpinar D.P., Balija M.B., Kügler S., Opazo F., Rezaei-Ghaleh N., Wender N. (2009). Pre-fibrillar α-synuclein variants with impaired β-structure increase neurotoxicity in Parkinson's disease models. EMBO J..

[45] Cheon M., Chang I., Mohanty S., Luheshi L.M., Dobson C.M., Vendruscolo M., Favrin G. (2007). Structural reorganisation and potential toxicity of oligomeric species formed during the assembly of amyloid fibrils. PLoS Comput. Biol..

[46] Orte A., Birkett N.R., Clarke R.W., Devlin G.L., Dobson C.M., Klenerman D. (2008). Direct characterization of amyloidogenic oligomers by single-molecule fluorescence. Proc. Natl Acad. Sci. USA.

[47] Carulla N., Zhou M., Arimon M., Gairí M., Giralt E., Robinson C.V., Dobson C.M. (2009). Experimental characterization of disordered and ordered aggregates populated during the process of amyloid fibril formation. Proc. Natl Acad. Sci. USA.

[48] Chatani E., Lee Y.H., Yagi H., Yoshimura Y., Naiki H., Goto Y. (2009). Ultrasonication-dependent production and breakdown lead to minimum-sized amyloid fibrils. Proc. Natl Acad. Sci. USA.

[49] Collins S.R., Douglass A., Vale R.D., Weissman J.S. (2004). Mechanism of prion propagation: amyloid growth occurs by monomer addition. PLoS Biol..

[50] Knowles T., Waudby C., Devlin G., Cohen S., Aguzzi A., Vendruscolo M. (2009). An analytical solution to the kinetics of breakable filament assembly. Science.

[51] Ecroyd H., Carver J.A. (2008). Unraveling the mysteries of protein folding and misfolding. IUBMB Life.

[52] Kumita J.R., Johnson R.J., Alcocer M.J., Dumoulin M., Holmqvist F., McCammon M.G. (2006). Impact of the native-state stability of human lysozyme variants on protein secretion by *Pichia pastoris*. FEBS J..

[53] Gill S.C., von Hippel P.H. (1989). Calculation of protein extinction coefficients from amino acid sequence data. Anal. Biochem..

[54] Han J.C., Han G.Y. (1994). A procedure for quantitative-determination of tris(2-carboxyethyl)phosphine, an odorless reducing agent more stable and effective than dithiothreitol. Anal. Biochem..

[55] Riddles P.W., Blakeley R.L., Zerner B. (1979). Ellman's reagent: 5,5′-dithiobis(2-nitrobenzoic acid)—a reexamination. Anal. Biochem..

[56] Serpell L.C., Berriman J., Jakes R., Goedert M., Crowther R.A. (2000). Fiber diffraction of synthetic α-synuclein filaments shows amyloid-like cross-β conformation. Proc. Natl Acad. Sci. USA.

