# Supplementary material for: The Non-Core Regions of Human Lysozyme Amyloid Fibrils Influence Cytotoxicity
Source: J Mol Biol. 2010 Oct 8;402(5-2):783–96. doi: 10.1016/j.jmb.2010.07.005 (PMC2954362; doi:10.1016/j.jmb.2010.07.005)
Supplement: Fig. S1 — Average length and length distribution of the FPhys and FAcid fibrils as analyzed from the TEM micrographs; Nfibril refers to the number of fibrils considered. Fibril solutions were examined using TEM by negative staining. Diluted samples were deposited onto Formvar-coated copper grids (400 mesh) (Agar Scientific, Stansted, UK). Filter paper was used to remove the excess of sample after 45 s, before adding a droplet of staining solution [2% (w/v) uranyl acetate]. After another 45 s, the excess was removed and the sample was left to air-dry. The micrographs were taken using a Transmission Electron Microscope JEOL 1010 operating at 80 kV. Images were taken with a Megaview III camera and digitized with the software AnalySIS (Soft Imaging System). TEM images were analyzed using ImageJ. From the TEM pictures, fibrils were counted and their length was measured to produce the size distribution of the fibrils. To obtain reliable statistics, between 150 and 230 fibrils were analyzed for each sample. [file mmc1.pdf]

Supplementary Material

The non-core regions of human lysozyme amyloid fibrils give rise to cytotoxicity

Maria F. Mossuto, Anne Dhulesia, Glyn Devlin, Erica Frare, Janet R. Kumita, Patrizia Polverino de Laureto, Mireille Dumoulin, Angelo Fontana, Christopher M. Dobson and Xavier Salvatella

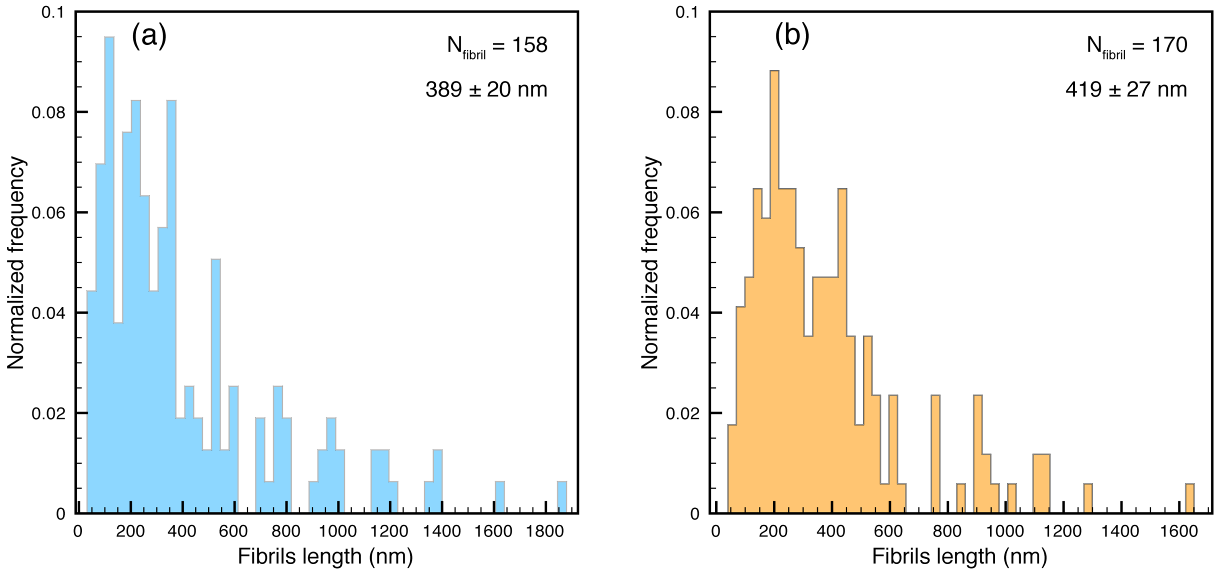

Fig S1: Average length and length distribution of the F^{Phys} and F^{Acid} fibrils as analyzed from the TEM micrographs; N_{fibril} refers to the number of fibrils considered.

Fibril solutions were examined using TEM by negative staining. Diluted samples were deposited onto Formvar-coated copper grids (400 mesh) (Agar Scientific, Stansted, UK). Filter paper was used to remove the excess of sample after 45 s, before adding a droplet of staining solution (2% (w/v) uranyl acetate). After another 45 s, the excess was removed and the sample was left to air-dry. The micrographs were taken using a Transmision Electron Microscope JEOL 1010 operating at 80kV. Images were taken with a Megaview III camera and digitized with the software AnalySIS (Soft Imaging System). TEM images were analysed using ImageJ. From the TEM pictures, fibrils were counted and their length was measured to produce the size distribution of the fibrils. To obtain reliable statistics, between 150 and 230 fibrils were analyzed for each sample.

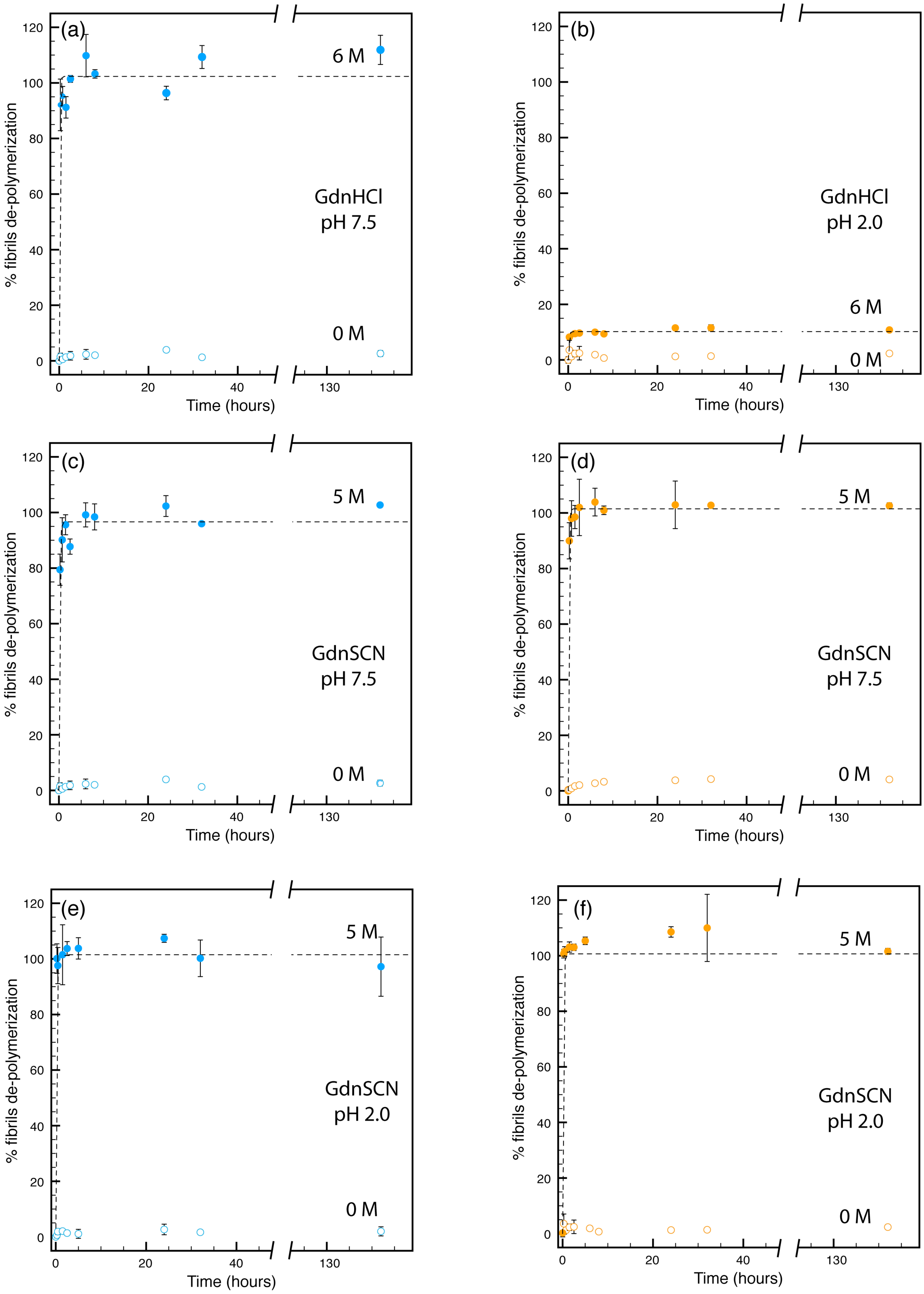

Fig S2: Depolymerization rate of F^{Phys} (blue) and F^{Acid} (orange) fibrils in presence of guanidine salts. The reactions were initiated by diluting fibrillar samples into buffered solutions of the chaotropic agent. At each incubation time the reaction mixture was analyzed by ultracentrifugation and the Bradford assay. Each point represents the average of three independent experiments.
